# Development of the *Together - Teens&20s* microsite, an online resource for adolescent and young adult cancer patients

**DOI:** 10.1016/j.pecinn.2023.100235

**Published:** 2023-11-20

**Authors:** Sarah Daniels, Elizabeth Bartholomew, Heather Chambliss

**Affiliations:** a Child Life Program, St. Jude Children's Research Hospital, 262 Danny Thomas Place, MS 121, Memphis, TN 38105, USA; b Department of Strategic Communications, Education, and Outreach, St. Jude Children's Research Hospital, Memphis, USA

**Keywords:** Website, Patient education, Adolescent and young adult (AYA), Cancer

## Abstract

**Objectives:**

The purpose of this study was to explore the information needs and preferences of adolescent and young adult (AYA) cancer patients to guide next steps for development of a new online resource for this audience. The goals were to understand information needs, prioritize topic areas, and inform website design and functionality.

**Methods:**

Participants were AYA cancer patients ages 13–25 years. We used multiple methods in two phases to gather audience feedback. The first phase collected quantitative data in an electronic survey (*n* = 45) about AYA patients' internet use and content preferences. The second phase collected qualitative data through focus groups (*n* = 13) about user preferences for content design and website function.

**Results:**

Survey results showed AYA patients were more interested in content about how treatments would impact their lives rather than the treatments themselves. They preferred content on school, careers, relationships, independence, side effects, and fertility. The focus groups indicated AYA patients prefer a clean, stylish design; infographics and visual aids; and the ability to find information quickly and control the amount they read.

**Conclusions:**

Online resources represent an important opportunity to advance cancer education for AYA patients. However, it is important to consider the information needs and preferences of this audience in content design and delivery.

**Innovation:**

Practitioners should use stakeholder input to guide the creation of patient education resources that are age- and audience-appropriate. This study provides insights into AYA information needs and preferences in pediatric oncology.

## 1 Introduction

Improved health literacy, health communication, and health information technology are recognized as important influencers of individual and public health that can impact health behavior, health equity, and health outcomes [1,2]. Availability and use of online health information have increased significantly in recent years. Most U.S. adults report using the internet to search for health information [3]. For many people, the internet is where they turn first for information about their disease, treatment, and care. For example, most participants in the Health Information National Trends Survey (HINTS) reported going to the internet first in their most recent search for health information [4].

Like other patient populations, patients and families facing cancer often turn to websites and online forums for health information. In addition, patients and caregivers express a desire to connect with others in a similar situation to share information and find support [5]. The *Together by St. Jude*™ website was created by St. Jude Children's Research Hospital (SJCRH) to serve as a comprehensive online resource for patient families facing pediatric cancer, no matter where the patient receives treatment. Website sections include About Pediatric Cancer, Diagnosis and Treatment, Care and Support, For Families, and Life After Cancer. About Pediatric Cancer includes information on different types of cancer; Diagnosis and Treatment covers topics including tests, procedures, cancer treatments, medicines, and side effects; Care and Support addresses supportive medical care topics and clinical areas such as rehabilitation, mental health, clinical nutrition, infection prevention, and palliative care; For Families includes psychosocial and daily living topics such as navigating health care, marriage and parenting, early childhood development, school support, spirituality, and bereavement; Life After Cancer provides information on survivorship, late effects, and physical and emotional well-being for cancer survivors. The website also has a video library and blog including patient and family stories and topics span cancer journey stages, from newly diagnosed to survivorship to bereavement.

Most recent website analytics reveal that, since its launch in 2018, the website has had over 9 million unique visitors from approximately 240 countries and territories. Although much of the content is written for the primary audience of parents and family caregivers of children with cancer, patients themselves represent key secondary target audiences for this resource. However, specific content geared toward adolescent and young adult (AYA) patients was lacking from the initial online resource.

Within the cancer patient population, AYA patients represent a distinct subgroup spanning the pediatric and adult oncology worlds. While defined as patients ages 15–39 years by the National Cancer Institute, the lower and upper age limits of AYA may vary by program and by institution [6,7]. At SJCRH, a pediatric institution, adolescent programming targets patients ages 13 years and older, with pediatric clinical trial eligibility often capping at 25 years old. Among this younger subgroup of AYA, there is a high proportion of childhood cancers including lymphomas, brain and nervous system tumors, acute lymphocytic leukemia, bone tumors, and soft-tissue sarcomas [8]. Many of these patients receive treatment in pediatric care settings.

For patients in their teens and early twenties, this developmental stage represents a time of increasing maturity and independence [9]. AYA patients may want to play a more active role in their care and treatment compared to younger age groups [10,11]. However, AYA patients can differ widely in their preferences, roles, and responsibilities around information exchange, decision making, advocacy, and care management [10,11]. The stages of adolescence and early adulthood have unique physical, social, emotional, and cognitive characteristics [8,9], all of which potentially impact how AYA learn and communicate about topics related to their care journey. A survey of Australian AYA patients found a high degree of unmet information needs including long term health effects, fertility, and risk of relapse or second cancers. Other relevant topics included financial concerns, sexuality, and wellness behaviors [12].

Digital technology and social media platforms have changed the information infrastructure, particularly for teens and young adults. According to a recent survey, 95% of U.S. teens report having access to a smartphone, and 90% have access to a desktop or laptop computer [13]. Almost all adolescents report daily use of the internet, with 46% reporting that they use the internet “almost constantly.” Social media accounts for much of the time that teens spend online, particularly YouTube, TikTok, Instagram, and Snapchat, platforms that are video and visual-based [13]. However, little is known about what AYA patients facing cancer or other serious illness want or need in an online health resource.

The purpose of this study was to explore the online information needs and preferences of AYA cancer patients to guide content development and delivery for this specific target audience. Goals were to: 1) identify and prioritize informational needs based on topic interest and content gaps; 2) inform website design; and 3) format content to appeal to the target audience.

## 2 Methods

A two-phased approach was used to gather feedback from individuals who represented potential users of an online pediatric cancer information resource specifically designed for AYA cancer patients. The first phase (February to April 2019) collected quantitative data via an electronic survey to assess informational needs and interests. The second phase (July and October 2019) collected qualitative data via focus groups to understand design and function preferences.

### 2.1 Participants

Participants were patients of SJCRH and affiliate clinics, anywhere from newly diagnosed through survivors of pediatric cancer. Following an ethics review, the Institutional Review Board provided approval and designated this study as non-human subjects' research. Participants were eligible for the study if they were aged 13–25 years, were diagnosed with a pediatric cancer, and were English-speaking. All participants completed a consent form prior to participation and were notified that participation was completely voluntary. All study participants were patients of SJCRH and affiliate clinics, anywhere from newly diagnosed through survivors of pediatric cancer. Following an ethics review, the Institutional Review Board provided approval and designated this study as non-human subjects' research. All participants completed a consent form prior to participation and were notified that participation was completely voluntary.

### 2.2 Data collection

#### 2.2.1 Survey

An electronic survey was created for this study. The purpose of the survey was to evaluate AYA patients' informational needs and preferences related to the impact of cancer on aspects of adolescent social, physical, and cognitive development. Questions assessed how AYA prefer to access technology (i.e., on their phone or another device), whom they prefer to receive cancer-related information from (i.e., health care providers or peers), and which topics they are interested in learning about In the survey, AYA patients were asked to rate their level of interest in specific topics related to four general domains of information: (1) social and relationship topics; (2) body image, appearance, and physical function; (3) school and work; and (4) medical and health. For each topic, AYA patients were asked to rate their level of interest using a 4-point scale, from *not at all interested* (1) to *very interested* (4). See Table 1 for survey topics and questions.

**Table 1:** Survey domains and topics.

Domain	Number of questions in domain	Topics *Participants were asked to indicate their level of interest:* *Not at all interested (1); Somewhat interested (2); Interested (3);* *Very interested (4)*
Social and relationship topics	11	• Friendships • Parents • Siblings • Dating • Marriage • LGBTQ • Online/ social media presence • Becoming more independent • Coping with loss • Leaving a legacy • Communicating with health care team
Body image, appearance, physical function	11	• Changes in weight • Hair loss • Scars • Amputation • Prosthesis/ splints • Using a wheelchair • Mobility limitations • Having a device (tube, line, etc.) • Eyeglasses/ vision changes • Lazy eye/ wearing eye patch • Hearing aids/ speech difficulties
School and work	16	• Keeping up with school • Types of academic support • Coping with changes in thinking and learning • Sports • Hobbies • Feeling left out • Bullying • College applications/ scholarships • Independent living • Personal finances • Talking with classmates/coworkers • Choosing a career • Workplace accommodations • Disability benefits • Health insurance • Access to medical care
Medical and health topics	12	• Medical information on specific cancers • Survival rates or prognosis • Procedures, scans, and tests • Treatments • Side effects • Late effects • Participating in medical decisions • News about research • Nutrition • Exercise • Rehabilitation therapy • Sexual health

Participants were recruited through the teen program at SJCRH and affiliate clinics in various ways. A flyer that included a QR code directed the participant to the survey. Flyers were posted on physical and electronic bulletin boards throughout the hospital on both inpatient and outpatient units and in clinical and non-clinical areas. An iPad station was also placed in hospital-designated teen rooms for targeted recruitment. Flyers and a survey link were also emailed to health care providers working with the target population both at SJCRH main campus and affiliate sites.

#### 2.2.2 Focus groups

In-person focus groups were conducted at the SJCRH main campus to assess factors related to website design and delivery preferences. Participants for the focus groups were referred to the project team by medical and psychosocial providers. A semi-structured question guide facilitated discussion on website navigation, appearance, function, and user preferences regarding cancer information. During the focus groups, participants were asked about their habits and preferences for seeking information online and shown examples of online content presented in different layouts and designs. AYA patients provided their input regarding content layout, page features, visual design, and other user experience topics. Focus groups were utilized rather than individual interviews to encourage collaborative conversation among AYA patients, where shared preferences and contrasting opinions could be identified and elaborated upon. Each session was moderated by the project team, including a medical writer, a graduate-level communications intern, and a Certified Child Life Specialist (CCLS). Participants were notified of the purpose and procedure of the focus group, including audio recording for the duration of the session. Participants were asked to provide verbal consent for their participation. Throughout each session, observer notes were recorded, and audio recording was used to document responses.

### 2.3 Data analysis

Survey responses were descriptively analyzed using IBM SPSS Statistics 25. Qualitative data from the focus groups were analyzed using inductive content analysis in accordance with the process described by Elo and Kyngäs [14]. The first author (SD) has expertise in leading qualitative research projects and oversaw the qualitative analytic process for this study. Training on qualitative coding procedures was provided to ensure consistency and quality of analysis among the study team. Audio recordings were made of each session, and they were transcribed verbatim. Transcripts were then inductively coded by two independent raters for recurring words and ideas. The study team met regularly to review and reconcile any discrepancies. The final codes were then organized into themes to represent the dataset. To ensure credibility of the qualitative findings, the researchers used iterative questioning throughout the focus group sessions and researcher understanding of the preferences shared by AYA participants was confirmed throughout the sessions [15]. Consensus of the final themes was reached following discussion by the study team.

## 3 Results

### 3.1 Survey

A total of 45 AYA patients, aged 13–25 years, completed the survey (*M =* 17 years, 51% female, 73% white). See Table 2 for demographic information of survey participants. 44% of respondents indicated they were leukemia or lymphoma patients with 44% indicating they were currently on therapy and 44% in remission. The first section of the survey asked participants to report about their general internet and social media use. Respondents reported using the internet and social media for at least 4–5 h daily. Respondents were also asked about which devices they most often use to access the internet, and nearly all AYA patients (96%) reported a preference for smartphone.

**Table 2:** Demographic information of survey participants (N = 45).

	Item	Count	Percentage
Gender			
	Male	22	48.9%
	Female	23	51.1%
Age			
	13–15 years	14	31.1%
	16–18 years	17	37.8%
	19–21 years	12	26.7%
	22–24 years	2	4.4%
Race/Ethnicity			
	White	34	75.6%
	Hispanic or Latino	8	17.8%
	Black or African American	4	8.9%
Medical Status			
	On-therapy	20	44.4%
	Newly Diagnosed	5	11.1%
	Long-term Survivor	4	8.9%
	Relapsed	2	4.4%
	In Remission	20	44.4%
Diagnostic Category			
	Leukemia/Lymphoma	25	55.6%
	Solid Tumor	12	26.7%
	Brain Tumor	8	17.8%

**Fig. 1:**
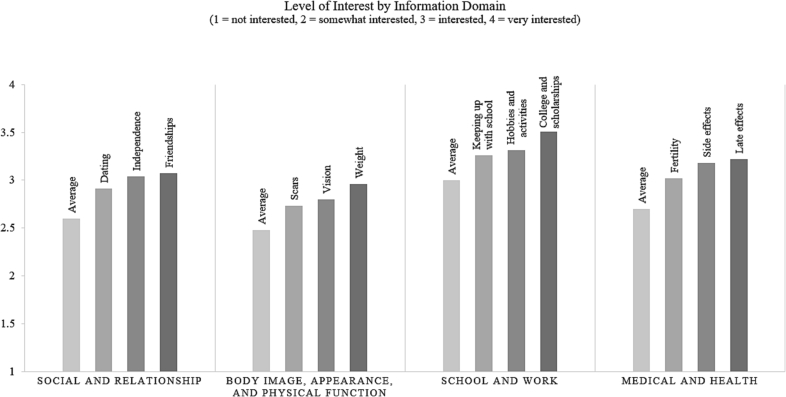
Average and top three topics by level of reported interest for each informational domain.

Descriptive analysis revealed key preferences among AYA participants. For the social and relationship domain, AYA patients were most interested in topics related to friendships, gaining independence, and dating. For the body image, appearance, and physical function domain, AYA patients reported highest interest in changes in weight, scars, and vision. For the school and work domain, AYA patients were most interested in topics related to college, hobbies and activities, and keeping up with school. Notably, the school and work domain pulled the highest interest scores overall. Lastly, in the medical and health domain, the top three topics of interest were late effects, side effects, and fertility. Respondents seemed more interested in how treatments would affect them as opposed to learning more information about the treatment itself. The average overall score for interest level across domains was 2.7, meaning AYA patients are at least somewhat interested in learning about those topics; this finding suggests that AYA are likely to utilize a site that addresses their informational needs. See Fig. 1 for interest level for each domain.

AYA patients were also asked about how they want to access information related to psychosocial and medical topics, separately. Regardless of the topic, AYA patients requested the same top three ways to get information: watching a video about the topic, watching a video about someone who has been through a similar experience, and reading an article. Interestingly, looking at an infographic and photographs were higher on the preference list for medical information compared to psychosocial topics, demonstrating that AYA patients have preferences for visual, concrete educational content when it comes to their medical experiences.

#### 3.1.1 Focus groups

A total of 13 AYA patients participated in the focus groups (group 1, *n* *=* 6; group 2, *n* *=* 7). See Table 3 for demographic information of participants. Three primary themes emerged about what AYA patients prefer from an online information resource: 1) bright, sophisticated colors that are clean, stylish, and have continuity in design, 2) infographic style for content presentation, and 3) ability to find information quickly with an option to control the amount they read.

**Table 3:** Demographic information of focus group participants.

Gender	Age	Race/Ethnicity	Diagnosis Category
Female	16	White	Leukemia
Female	15	White	Leukemia
Female	20	White	Thyroid
Male	21	White	Solid Tumor
Male	18	White	Leukemia
Female	15	White	Brain Tumor
Female	22	Hispanic	Leukemia
Male	16	White	Brain Tumor
Female	17	White	Leukemia
Female	20	Hispanic	Leukemia
Female	19	Hispanic	Lymphoma
Female	19	White	Leukemia
Female	15	White	Solid Tumor

##### 3.1.1.1 Theme 1: AYA patients prefer a bright, clean, stylish design

AYA cancer patients shared opinions and preferences regarding visual design of online content. Visual design focuses on the aesthetics of a site and the strategic implementation of images, colors, fonts, and other elements. A successful visual design enhances the content on the page by engaging users and helping to build trust and interest. AYA cancer patients have a clear preference for bright, sophisticated colors and a clean, stylish design. Participants reported wanting bright, colorful designs because they are more eye-catching. The consistency of a theme was equally meaningful for user experience. Once a theme is chosen, it is important to stick to it, as participants noticed when graphics did not fit well together. For instance, one participant noted a preference for a strategic, discriminating choice of colors: “I also like the theme of the colors. They're not just doing every color in the rainbow. They're keeping consistent. It'd be too much if it was like every color” (female, 22, leukemia). It was also important to AYA patients that theme is not too childish. One participant noted, “I hate when people put teenager design and then they put emojis all over it. And I think, ‘you tried’” (female, 17, leukemia). AYA patients wanted a space designed just for them.

##### 3.1.1.2 Theme 2: AYA patients prefer infographics and visual aids

For content presentation, AYA patients indicated a preference for infographics. An infographic is a chart, diagram or illustration that uses graphic elements to present information in a visually striking way. Participants' first impression of the infographic format was that it was overwhelming. However, they felt more comfortable with the information presented after viewing infographics. The infographic did not have too many words. The visuals helped get the message across in a comprehensive way: “I like reading graphics like this because it tells you everything you need to know” (female, 20, thyroid cancer). Importantly, visuals also seemed to help AYA cancer patients digest complex information in new ways: “It's just like the little graphics are cool. Even if you don't understand it, you can still visualize it” (male, 20, osteosarcoma). AYA patients agreed visual elements are key to comprehending information about cancer.

##### 3.1.1.3 Theme 3: AYA patients desire an ability to find information quickly and to control the amount they read

Participants preferred articles divided into smaller sections. They expressed that they do not like having to read through a whole page to get to desired information. AYA patients prefer to control the amount of information they receive. All participants reported wanting short articles with “read more” options. They indicated a concise introduction to the topic acts as a hook to get them to click “read more” to access more in-depth information. They also preferred a plus sign or a ‘Read More’ button that takes the reader to another page opposed to a direct link because it is a more concise method for getting to the next information. AYA patients enjoyed the experience of being able to read more because: “It's kind of like hooking you in you hear about something. It's like trying to tell a story and not telling you the whole story. You want to find out more about it. Like you can click on the plus signs and link to other places along with the facts that are in there and get more in-depth” (female, 16, leukemia). AYA patients preferred interactive content navigation and ability to seek more information on a topic to control the level of information they received.

#### 3.1.2 Teens&20s microsite

The findings from this study were used to guide the creation of a microsite called Teens&20s on the *Together by St. Jude*™ resource. A microsite is an individual web page or set of pages that function as a separate entity within a larger website. Survey results were used to identify AYA patients' preferences regarding informational content and key topics. Focus group results were utilized to further inform the content design elements and interactive features of the platform. See Table 4 for examples of how the results from the survey and focus groups informed content development and delivery of the Teens&20s microsite. Next steps for this research will be to evaluate microsite performance over time.

**Table 4:** Examples of How Input from AYA Patients Informed Content and Design of Together Teens&20s.

Survey Finding	Focus Group Finding	Implementation
Sections determined by topic preferences of AYA participants.	“They're more colorful, bright, they draw your eye to it.” (15-year-old female with brain cancer) “It's just a clean design. It overall looks nice.” (20-year-old male with osteosarcoma)	Bright colors, and common theme
Preference for infographics	“I like reading graphics like this because it tells you everything you need to know.” (20-year-old female with thyroid cancer) “It's just like the little graphics are cool. Even if you don't understand it, you can still visualize it” (20- year-old male with osteosarcoma)	
Preference for an option to control the amount of information they receive while they navigate the site.	“It kind of makes you want to know since it has extra buttons it makes you want to find out more because it only tells you a little.” (15-year-old female with brain cancer) “It's kind of like hooking you in you hear about something. It's like trying to tell a story and not telling you the whole story. You want to find out more about it. Like you can click on the plus signs and link to other places along with the facts that are in there and get more in-depth.” (16-year-old female with leukemia)	

## 4 Discussion and conclusion

### 4.1 Discussion

This study explored AYA cancer patients' preferences for the development and delivery of web-based health information. Through surveys, AYA patients were invited to share about their feedback regarding both the type of information that they need and how content should be delivered. In focus groups, AYA patients provided deeper insights about content design and delivery features to promote user experience and engagement for AYA audiences. These insights informed the creation of a cancer education microsite for AYA patients based on audience feedback.

AYA patients and families collaborate throughout the treatment trajectory to consume relevant information and participate in care decisions alongside health care providers ^10^. To fully engage in this process, it is important that AYA patients have access to accurate and relevant medical and psychosocial patient education. Access to audience-appropriate information can motivate AYA cancer patients to become more active participants in their care, an opportunity that supports the developmental task of autonomy [12]. However, AYA patients and families often report that they have high levels of unmet information needs covering both medical and psychosocial topics [12,16]. Unfortunately, a limited knowledge base related to cancer and general medical care makes AYA patients unprepared for the realities of treatment and less likely to readily identify symptoms and seek help [17]. In addition, AYA patients report receiving inadequate information regarding certain topics such as late effects from treatment, which could impair decision making [18]. Health care practitioners are not always aware of how much information AYA patients want and need [19]. Online sources of health information may supplement and enhance in-person conversations by helping AYA patients explore and identify information needs for further discussion with their care team.

Findings from the current study expand on current literature about the information seeking preferences of AYA patients, particularly on online platforms. In addition to supporting the role of AYA patients as participants in their own care, the perspectives shared here also reveal specific suggestions for developing and delivering relevant information to AYA with cancer.

AYA patients in the current study reported a higher interest in accessing content related to psychosocial topics than medical topics. This supports findings that many AYA patients feel they receive high quality medical information following diagnosis, but might struggle meeting social, behavioral, or more complex informational needs [16,18,19]. AYA patients in the current study were most interested in topics related to school, adulting, side effects and late effects, healthy living, emotional health, and personal stories from peers with similar experiences. Importantly, higher interest topics tended to be future directed, or self-care focused.

These preferences align with the idea that cancer is just one part of an AYA patient's life and is not all-encompassing [21]. True, cancer is disruptive in many ways, but AYA patients are in a developmental period characterized by their many aspects of identity [9,22,23]. Therefore, a focus on relationships, vocational aspirations, and hobbies are significant [24].

In considering the best ways to meet the information needs of AYA patients, the delivery source and design of content are aspects that should be given attention. Online sources are of particular interest for AYA patients seeking health behavior information [20], and online delivery provides important opportunities to improve access and increase engagement with education and support resources. Following an Institute of Medicine (IOM) workshop and review of gaps in AYA cancer care, experts highlighted the need to improve patient education for this patient population with a specific call to develop and leverage online resources to communicate with AYA patients [21]. Indeed, internet use is associated with younger age as is seeking information about cancer from sources beyond practitioners [25]. A recent survey of AYA treated for cancer found that most patients searched the internet for information several times per week after diagnosis and daily to several times per week during treatment [19].

Results from this study suggest practical recommendations in designing health-related education specifically for AYA patients. AYA patients requested an online space to call their own and a responsive platform to provide more control of information. These preferences align with general AYA psychosocial development including a desire to distinguish themselves from others and to have autonomy over their actions. Information seeking is a coping strategy for AYA patients [25] because it is a way to gain control, something important to this life stage. But, information seeking can be overwhelming and not all patients want the same level of information [25]. This sentiment is embodied in AYA patients' statements that they want bite-sized information with an option to click to “read more”. This level of control over information seeking is something that AYA patients may not be offered in-person contexts. In addition, bright colors and stylish layout were suggestions that help AYA patients distinguish their platform from others directed at children or adults. AYA patients reported being most drawn to visual designs compared to written information. Infographics, photography, and video were most wanted features, especially for medical information. These features may be more interactive and engaging for AYA patients.

The current study was exploratory and used a quality improvement framework. Given the research design and setting, several limitations should be considered. First, the goal was to inform next steps to expand educational content provided on a web-based pediatric oncology resource for patient families, and the focus was limited in terms of disease types, topics addressed, and patient age. In addition, participants were recruited from one treating institution and did not represent all cancer diagnoses or treatment trajectories. Future research examining informational needs and preferences of AYA audiences could consider examining how results might vary by age ranges, health status, or diagnoses, including illnesses other than cancer. Additionally, it is important to note that participants were predominantly White females from one treating institution. Given that this resource is intended for a global audience, future studies could recruit a more diverse group of patients including non-English speakers. Results of this research informed the *Together by St. Jude*™ Teens&20s microsite, including the prioritization of topics, information architecture, and visual design; however, the effectiveness of the content and delivery has not been fully evaluated. Future investigation could include user testing to examine how well the website is meeting audience needs. Additional studies are needed to understand how online content and technology features impact usability and learning outcomes as well as self-efficacy, decision making, and patient-provider communication.

### 4.2 Innovation

This study contributes to the innovation of patient education research for AYA by demonstrating the value of using a patient-centered research design involving multiple methods for data collection. We used a user-centered design to inform next steps in the development of a web-based educational resource tailored specifically to AYA cancer patients treated in a pediatric cancer setting. By combining survey and focus group methodology, we were able to identify the online information needs and preferences of this audience as well as explore design features to enhance appeal. This approach enabled AYA to communicate their preferences in a variety of ways, giving them control over how to express the nuances of their views about what and how information should be presented in an online resource.

This project also contributes to the evidence base highlighting the significance of an audience-first approach. Early engagement of the AYA target audience enables the prioritization of topics that are most meaningful to this specific patient population. There are likely to be discrepancies between what AYA identify as high priority informational needs as compared to what their parents or health care professionals prioritize. Through focus groups, we were able to hear from patients directly regarding what they wanted in an online resource designed specifically for them. The use of non-medical website examples further prompted feedback and discussion among participants. An overall impression emerging from the survey and focus group themes is the idea that the AYA patient wants to be seen as more than their diagnosis, fitting with the developmental stage of adolescence. Similarly, AYA are typically avid users of technology and prefer a stylish design and use of video content.

A final novel contribution of this work is the insight it provides to the AYA patient population treated in a pediatric setting. Many AYA are treated in adult settings and are provided with adult-centered information about their diagnosis. In pediatric settings, AYA may be overlooked when health care professionals share information with caregivers. This study emphasizes the AYA patient as living in a unique and distinctive developmental period, deserving of attention to their age-specific needs. It is essential that future studies focus on the AYA population rather than group them with either pediatrics or with adults.

### 4.3 Conclusion

Recent years have seen a rapid transition of health information and patient education to digital format and delivery. Considering AYA patients' high internet use, especially on mobile devices, as well as their developing sense of independence and desire for autonomy, online resources represent an important opportunity to advance patient education for this population. To increase access, use, and effectiveness, educational resources should be designed to be age- and audience-appropriate by leveraging guidance from stakeholder feedback.

## Disclosures

The authors do not have any real or perceived conflicts of interest to disclose.

## Funding

This research did not receive any specific grant from funding agencies in the public, commercial, or not-for-profit sectors. Support to St. Jude Children's Research Hospital was provided by the American Lebanese Syrian Associated Charities (ALSAC).

## CRediT authorship contribution statement

**Sarah Daniels:** Writing – review & editing, Writing – original draft, Visualization, Supervision, Project administration, Methodology, Formal analysis, Data curation, Conceptualization. **Elizabeth Bartholomew:** Writing – review & editing, Writing – original draft, Visualization, Supervision, Formal analysis, Data curation, Conceptualization. **Heather Chambliss:** Writing – review & editing, Writing – original draft, Visualization, Formal analysis, Data curation, Conceptualization.

## Declaration of Competing Interest

The authors of this article have no financial or personal competing interests to declare that influenced the work shared in this paper.

## References

[1] U.S. Department of Health and Human Services (2020). https://wayback.archive-it.org/5774/20220413183358/https://www.healthypeople.gov/2020/topics-objectives/topic/health-communication-and-health-information-technology.

[2] U.S. Department of Health and Human Services. Health Communication - Healthy People 2030 Health.Gov. n.d. Accessed April 4, 2023 https://health.gov/healthypeople/objectives-and-data/browse-objectives/health-communication.

[3] Pew Research Center N (2023). https://www.pewresearch.org/internet/2013/02/12/the-internet-and-health/.

[4] Bangerter L.R., Griffin J., Harden K. (2019). Health information–seeking behaviors of family caregivers: analysis of the health information National Trends Survey. JMIR Aging.

[5] Elsbernd A., Crenner C., Rosell T. (2019). Individual experiences and utilization of supportive resources in adolescents and young adults with cancer. J Adolesc Young Adult Oncol.

[6] Osborn M., Johnson R., Thompson K. (2019). Models of care for adolescent and young adult cancer programs. Pediatr Blood Cancer.

[7] Ferrari A., Stark D., Peccatori F.A. (2021). Adolescents and young adults (AYA) with cancer: a position paper from the AYA working Group of the European Society for medical oncology (ESMO) and the European Society for Paediatric Oncology (SIOPE). ESMO Open.

[8] Coccia P.F., Pappo A.S., Beaupin L. (2018). Adolescent and young adult oncology, version 2.2018, NCCN clinical practice guidelines in oncology. J Natl Compr Cancer Netw.

[9] Steinberg L.D. (2015).

[10] Sisk B.A., Keenan M., Kaye E.C. (2022). Co-management of communication and care in adolescent and young adult oncology. Pediatr Blood Cancer.

[11] Sisk B.A., Keenan M., Schulz G.L. (2022). Interdependent functions of communication with adolescents and young adults in oncology. Pediatr Blood Cancer.

[12] McCarthy M.C., McNeil R., Drew S. (2018). Information needs of adolescent and young adult cancer patients and their parent-carers. Support Care Cancer.

[13] Pew Research Center (2022). Teens, Social Media and Technology 2022. https://www.pewresearch.org/internet/2022/08/10/teens-social-media-and-technology-2022/.

[14] Elo S., Kyngäs H. (2008). The qualitative content analysis process. J Adv Nurs.

[15] Shenton A.K. (2004). Strategies for ensuring trustworthiness in qualitative research projects. Educ Inf.

[16] Kent E.E., Smith A.W., Keegan T.H.M. (2013). Talking about cancer and meeting peer survivors: social information needs of adolescents and young adults diagnosed with cancer. J Adolesc Young Adult Oncol.

[17] Hart R.I., Cowie F.J., Jesudason A.B. (2021). Adolescents and young adults’ (AYA) views on their cancer knowledge prior to diagnosis: findings from a qualitative study involving AYA receiving cancer care. Health Expect.

[18] Greenzang K.A., Fasciano K.M., Block S.D. (2020). Early information needs of adolescents and young adults about late effects of cancer treatment. Cancer..

[19] van de Graaf D.L., Vlooswijk C., Bol N. (2023). AYAs’ online information and eHealth needs: a comparison with healthcare professionals’ perceptions. Cancer Med.

[20] Pugh G., Hough R.E., Gravestock H.L. (2017). The health behavior information needs and preferences of teenage and young adult cancer survivors. J Adolesc Young Adult Oncol.

[21] Nass S.J., Beaupin L.K., Demark-Wahnefried W. (2015). Identifying and addressing the needs of adolescents and young adults with cancer: summary of an Institute of Medicine Workshop. Oncologist.

[22] Erikson E.H. (1994).

[23] Arnett J.J. (2000). Emerging adulthood: a theory of development from the late teens through the twenties. Am Psychol.

[24] Zebrack B., Isaacson S. (2012). Psychosocial care of adolescent and young adult patients with cancer and survivors. J Clin Oncol.

[25] Petersen E., Jensen J.G., Frandsen T.F. (2021). Information seeking for coping with cancer: a systematic review. Aslib J Inf Manag.

